# Differential impact of various reactive oxygen species (ROS) on HIF-1α/p53 direct interaction in SK-N-MC neuroblastoma cells

**DOI:** 10.1186/s13578-017-0180-4

**Published:** 2017-10-10

**Authors:** Elham Parandavar, Razieh Yazdanparast

**Affiliations:** Institute of Biochemistry and Biophysics, University of Tehran, P.O. Box 13145-1384 Tehran, Iran

**Keywords:** HIF-1α, p53, Hypoxia, Interaction, Apoptosis

## Abstract

**Background:**

A vital property of eukaryotic cells physiology is their rather quick response to variation of oxygen tension, mainly by a transcription factor known as hypoxia-inducible factor-1 (HIF-1). Aside from its transcriptional regulation, other mechanisms, such as post translational modifications and protein–protein interactions, the interaction between HIF-1α and p53 has attracted more attention mainly due to simultaneous enhancement in the protein levels of these two anti- and pro-apoptotic vital transcriptional factors within the ROS-stressed cells.

**Methods:**

In this study, we measured cell viability following exposure of the cells to H_2_O_2_, menadione and Cobalt Chloride by MTT, and ROS content was measured under the same condition. The immunoblotting technique has been used to establish the presence and amount of Caspase, HIF-1α and p53 proteins. Then, the effect of different ROS on interaction between HIF-1α and p53 proteins was examined by co-immunoprecipitation.

**Results:**

The results showed that cells viability and intracellular ROS content were modulated in response to menadione, H_2_O_2_ and Cobalt Chloride. These agents had different influence on HIF-1α signaling pathways as well as its interactions with p53 protein. It appeared that direct communication between HIF-1α and p53 proteins by ROS stresses, under both normoxic and hypoxic conditions, was governed by HIF-1α at a certain induced level.

**Conclusions:**

Our data indicated that stabilization, a prerequisite for communication, of HIF-1α is dependent to the types of free radicals.

## Background

All sorts of life rely on molecular oxygen within a narrow range of its tension. Variation in the intracellular level of oxygen has been associated with various physiological consequences naming oxidative stress resulting from surplus level of O_2_ (hyperoxia), ischemia and cellular demise arising from moderate-to-severe level of O_2_ depletion (hypoxia–anoxia) [1–3]. In general, physiological responses to hypoxia are categorized either as acute (quick-onset with short duration) or chronic (delayed-onset with prolonged duration). Acute responses are believed to be associated with variation in the activity of the existing proteins while, chronic responses are believed to entail variation in the expression level of the affected target genes [2, 4].

One of the well-studied factors induced in response to hypoxia is hypoxia inducible factor 1 (HIF-1), a heterodimeric protein composed of two subunits of HIF-1α and HIF-1β (also known as aryl hydrocarbon nuclear receptor transporter, ANRT). Both subunits belong to basic Helix-Loop-Helix per/Arnt/Sim (bHLH-PAS) transcription factor family [5, 6]. Apparently, HIF-β is constitutively expressed independent of the level of molecular oxygen and thus, it is a stable protein regardless of the physiological stresses. However, if not the expression level, but the stability of the HIF-1α subunit is regulated by the O_2_ level [7, 8]. In other words, HIF-1α acts as the O_2_-sensor and the regulatory sub unit of HIF-1 complex.

Despite the well accepted survival function of HIF-1α under mild hypoxia, recently it has been shown that HIF-1α trans-activate some of the pro-apoptotic genes such as NIP_3_, NIX, RT801 and P27 implying that HIF-1α is also involved in cell demise under oxygen-stressed environment [4, 8, 9]. These dual behaviors have in part been contributed to HIF-1α direct and/or indirect interaction with other proteins including p53 with as yet debatable physiological significance.

Under normoxia, the intracellular p53 and HIF-1α contents are low. Under this resting condition, p53 is subjected to Mdm2 ubiquitination and proteasomal degradation. However, under tight stress situation such as DNA damage or hypoxia, the ATM/ATR-dependent phosphorylation of p53 leads to its polymerization, p300 binding followed by its transcriptional activation [9, 10]. On the other hand, O_2_-dependent regulation of HIF-1α is believed to be mediated through its O_2_-dependent degradation domain (ODD) containing two regulatory proline residues. Under normoxia, hydroxylation of Asn 803 residue of HIF-1α by HIF-1α hydroxylase prevents p300 binding to HIF-1α. Besides, hydroxylation of prolines 402 and 546 within the ODD domain by proline hydroxylases (PHDs) initiates the binding of von Hippel–Lindou (pVHL) protein resulting in HIF-1α ubiquitination followed by proteasomal degradation. Under hypoxia, HIF-1α-hydroxylase and PHDs are inactive leading to HIF-1α accumulation, nuclear translocation followed by HIF-1β and p300 bindings [4] with the final outcome of HIF-1α stabilization and transcriptional activation. In other words, under moderate to severe hypoxia both p53 and HIF-1α are stabilized via competition for p300 co-activator which normally exists at minute amounts. Which one of these transcriptional factor succeed the competition would depend on the extent of oxygen-dependent stabilization of each of these two cellular adaptation factors. It is generally accepted that p53 stabilization occurs at severe hypoxic environment ([O_2_] < 1%) while, HIF-1α accumulation happens at much higher O_2_ level ([O_2_] > 3%).

The aforementioned mutual effect among p53 and HIF-1α might be due in part to the direct physical interaction between the two HIF-1α natively unfolded motifs within its acidic ODD domain with the basic DNA binding core of p53 [11, 12] and/or to the indirect interaction between HIF-1α and p53 via Mdm2 [13]. Anyhow, these interactions could bring about either transcriptional inactivation of p53, due to the masking of p53 DNA binding core by HIF-1α, leading to lower level of apoptosis or it could cause p53 transcription activation mainly due to the higher level of p53 binding to the co-activator, p300, and thus, higher level of apoptosis. Of note, some studies have shown that hypoxia-induced p53, albeit binding to target gene promotors, is incapable of trans-activating them [14]. In support of the former view, recently it has been shown that under hypoxia, Jab1 (Jun activation domain binding protein 1) competes with p53 for binding to HIF-1α leading to its stabilization and enhanced survival [15].

Despite the continuing debate on the physiological consequences of HIF-1α/p53 interactions and regarding the role of oxygen tension in ROS production and the proven role of ROS in HIF-1α stabilization [16], in this investigation we wished to evaluate modulation, if any, of HIF-1α and p53 direct interaction by the types of oxygen free radicals (ROS) mainly hydroxy free radicals (ȮH) and superoxide anion radicals (${\dot{\text{O}}}_{ 2}^{ - }$) whose intracellular contents are augmented under hypoxia [16].

## Results and discussion

### Cell viability modulation by the oxidants

**Fig. 1:**
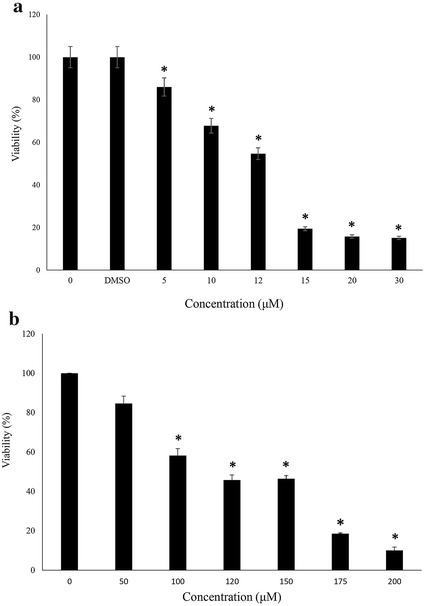
Effects of menadione and $\text{H}_{2} \text{O}_{2}$ on SK-N-MC cell viability. The cells (2.5 × 10^4^) 12 h after seeding have been exposed to various concentrations of Menadione **a** or ${\text{H}}_{2} {\text{O}}_{2}$ **b** for 24 h, followed by measuring the cells viabilities relative to untreated control cells. The data represent the mean ± SD of at least 3 independent measurements. Asterisk significantly different from control cells (p < 0.05)

It is well established that menadione and H_2_O_2_ induce oxidative stress in the exposed cells via superoxide anion and hydroxy free radicals, respectively [17]. Exposure of SK-N-MC cells to various doses of menadione (0–30 μM) and H_2_O_2_ (0–200 μM) affected the viability of the cells dose-dependently relative to un-exposed control cells as evident from Fig. 1a, b. Based on this figure, the IC_50_ doses were found to be around 10.96 and 107.61 μM for menadione and H_2_O_2_, respectively.

### Estimation of the intracellular ROS levels generated by the oxidants

**Fig. 2:**
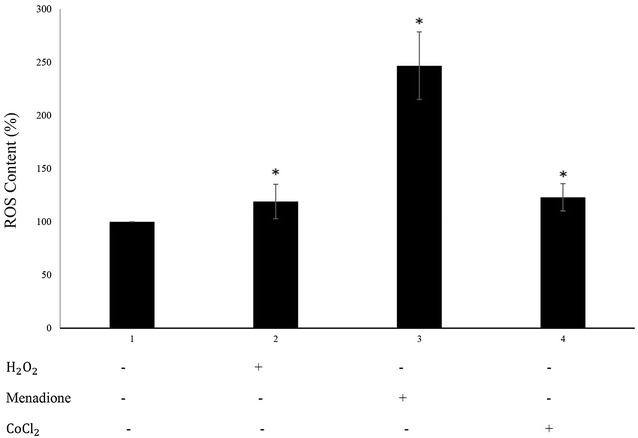
The interacellular ROS content of the oxidant-treated SK-N-MC cells. The cells were treated with either ${\text{H}}_{2} {\text{O}}_{2}$ (100 μM), menadione (12 μM) or CoCl_2_ (100 μM) for 24 h and then the ROS content was measured using DCFH-DA probe. The result are the mean ± SD of three experiments. Asterisk significantly different from the untreated control cells (p < 0.05)

The intracellular ROS content of each treated SK-N-MC cell sample was assessed by 2′,7′-dichloro fluorescein diacetate (DCFH-DA). Proportional to the intracellular ROS content, DCFH is oxidized to 2′,7′-dichloro fluorescein (DCF) which is a fluorophore and a means of quantification of the ROS content. As shown in Fig. 2, the SK-N-MC intracellular ROS content enhanced by 2.46- and 1.30-fold among the cells exposed for 24 h to the IC_50_ concentration of menadione (12 μM) and H_2_O_2_ (100 μM), respectively. Interestingly, CoCl_2_, as a hypoxia-inducing agent and as an oxidant also enhanced the ROS content of the cells at its IC_50_ (100 μM) by almost 1.6-fold (Fig. 2) and in accordance to the present literature [18–20].

### Apoptosis induction in SK-N-MC cells by the oxidants

**Fig. 3:**
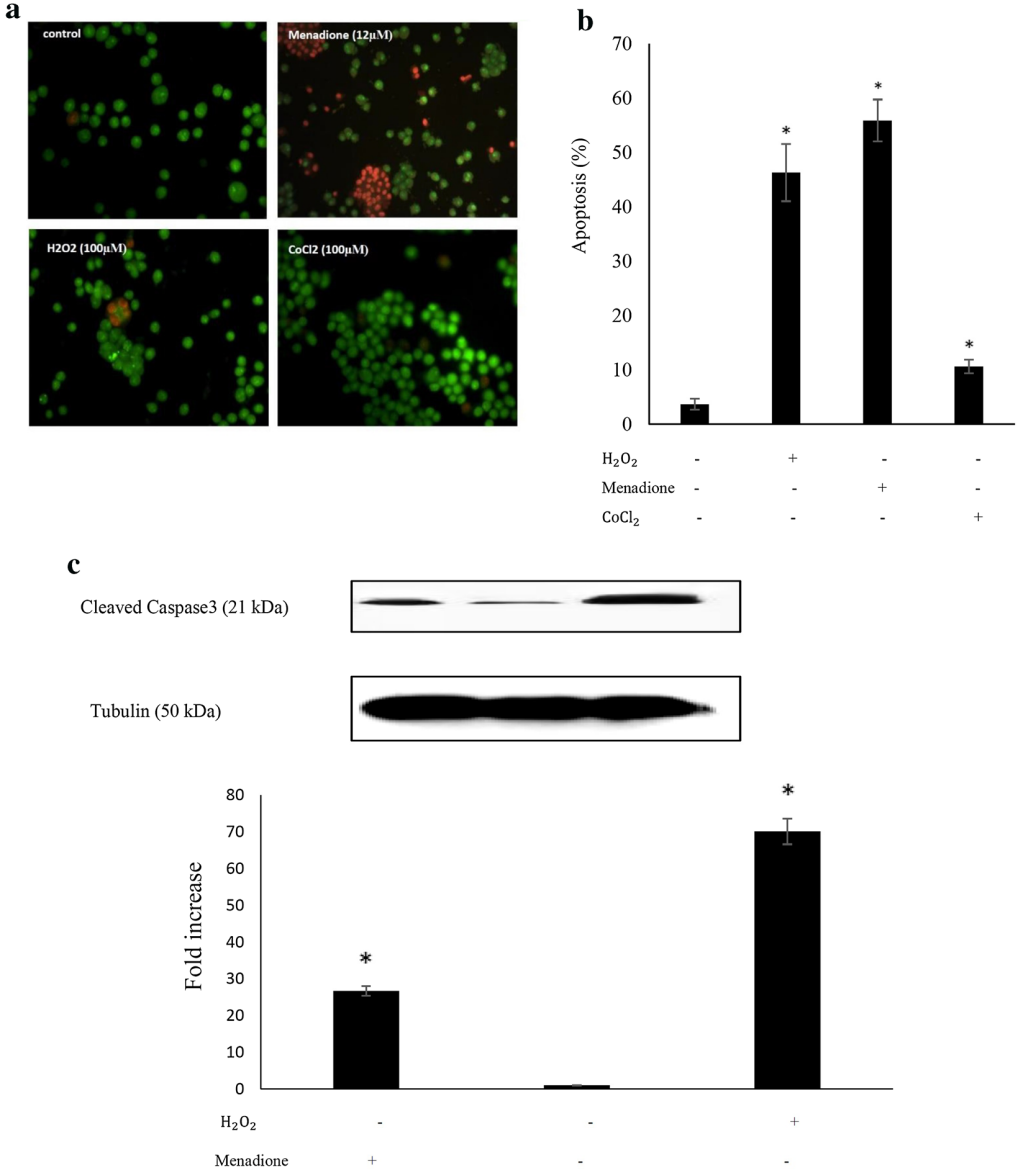
Evaluating apoptosis in SK-N-MC cells. **a** Cells were treated with 100 μM H_2_O_2_, 12 μM menadione and 100 μM CoCl_2_ for 24 h and then cells were visualized with Ethidium bromide/acridine orange dual-staining method. **b** The extent of apoptosis was determined based on counting apoptic cells in ten randomly chosen region by fluorescence microscope. **c** Caspase3 generation, as an apoptotic marker under oxidative stress condition, was analyzed using western blot technique. Asterisk significantly different from untreated control cells (p < 0.05)

As shown in Fig. 3a, b, following 24 h of exposure of the cells to H_2_O_2_ (100 μM) and/or Menadione (12 μM), the number of apoptotic cells increased from 3.60% in the untreated control cells to 46.30 and 40.88% in a caspase3-dependant manner (Fig. 3c), respectively. Despite the higher level of ROS generation in CoCl_2_-treated cells (100 μM) relative to H_2_O_2_-treated ones (Fig. 2), the extent of cell apoptosis was lower, by almost a factor of six, relative to the extent of apoptosis among the H_2_O_2_-treated cells. This response might in part be due to the types of reactive free radicals generated within the cells in response to stimuli. Physiologically, ROS (mainly ${\dot{\text{O}}}_{2}^{ - }$, ȮH and OONȮ) are primarily produced as superoxide anions within the inner membrane space of mitochondria, its matrix and to a lesser extent on the outer mitochondrial membrane. While the majority of ${\dot{\text{O}}}_{2}^{ - }$ within the matrix is converted to ${\text{H}}_{ 2} {\dot{\text{O}}}_{ 2}$ by the mitochondrial superoxide dismutase, part of ${\dot{\text{O}}}_{2}^{ - }$ produced in the inner membrane space leaks to the cell cytosol where it is converted to H_2_O_2_ by the cytosolic SOD. In addition, it has been shown that high ${\dot{\text{O}}}_{2}^{ - }$ level will act as an oxidant of [4Fe–S] cluster-containing proteins leading to ȮH production from H_2_O_2_ by making Fe^2+^ ion available for the Fenton reaction [21]. Hydroxyl free radicals, in turn, could react with nitric oxide (NO) to form peroxynitrite radicals (OONȮ), both being highly reactive and toxic to the biological systems [22]. Cellular peroxisomes are other site of H_2_O_2_ (but not ${\dot{\text{O}}}_{2}^{ - }$) generation. However, most of H_2_O_2_ is converted to H_2_O and O_2_ by the peroxisome catalase. Thus, ROS production within the cells could be either mitochondria-dependent, as it is the case in hypoxia, or it can be mitochondria-independent as in the case of CoCl_2—_induced ROS production. This situation might bring about differential cellular responses as it will be shown in the coming sections.

### HIF-1α stabilization in response to the types of oxygen free radicals

Hypoxic-inducible factor (HIF-1) is known as a master regulator of a vast array of genes in response to intracellular oxygen concentration. The regulatory subunit of HIF-1 complex is HIF-1α which is degraded under normoxic oxygen concentration of 21% but it is stabilized at hypoxic (3%) and anoxic (< 0.1%) oxygen level as a means of cellular adaption to stress [23, 24]. It has also been shown that other oxygen-independent mechanisms such as mitochondrial-dependent ROS production at low [O_2_] could lead to HIF-1α stabilization [16, 25, 26]. This latter mode of HIF-1α stabilization could be prevented by antioxidants such as NAC and ascorbate while hypoxic stabilization of HIF-1α is not affected by the same compounds, clearly implying that at low level of O_2_ tension, HIF-1α stabilization occurs via ROS while hypoxic stabilization is regulated by molecular oxygen. Regarding the proven role of ROS on HIF-1α accumulation, we compared the extent of HIF-1α stabilization by various types of oxygen free radicals under the influence of menadione (${\dot{\text{O}}}_{2}^{ - }$ generator), H_2_O_2_ (${\dot{\text{O}}\text{H}}$ generator) and CoCl_2_ (a hypoxic mimetic and ȮH and ONOȮ generator) at the IC_50_ concentration level of each compound.

**Fig. 4:**
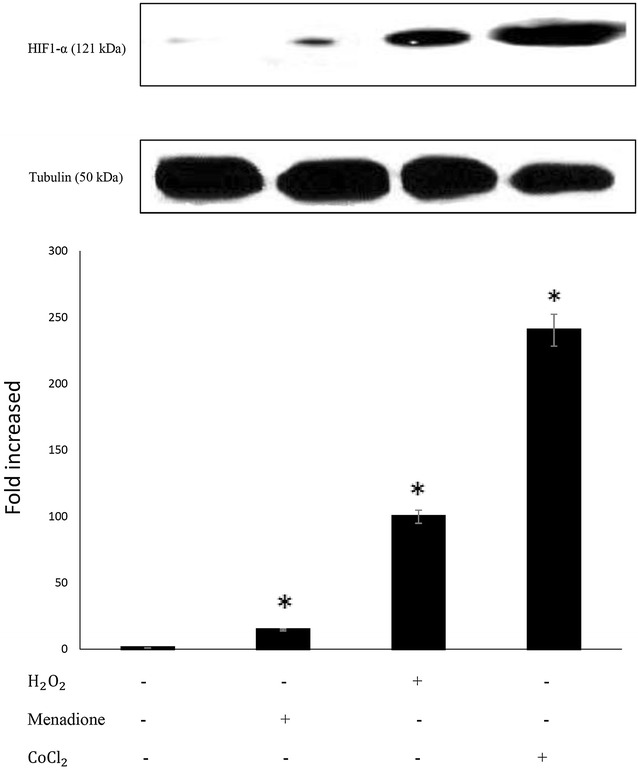
HIF1-α stabilization by manadione, H_2_O_2_ and CoCl_2_. SK-N-MC cells were treated with 100 μM H_2_O_2_, 12 μM menadione and 100 μM CoCl_2_ for 24 h. Protein expression was analyzed by Western blot technique. The intensity of each band was examined using densitometry analyses and the values were normalized with respect to B-tubulin and are shown relative to control. The histogram values represent the mean ± SD for 3 independent experiments. Asterisk significantly different from control cells (p < 0.05), one prototype of western blot is shown

Based on western blot analyses as shown in Fig. 4 (top), the three types of free radicals were capable of stabilizing HIF-1α though to different extent after 24 h of exposure. As evident from Fig. 4 (bottom), CoCl_2_ at 100 μM, which is believed to induce hypoxic environment [27, 28], caused the highest HIF-1α accumulation relative to menadione at 12 μM and/or H_2_O_2_ at 100 μM which have been applied at normoxic condition. From this observation, it could be concluded that higher HIF-1α stabilization occurs at lower oxygen tension.

### p53 stabilization in response to the type of oxygen free radicals

**Fig. 5:**
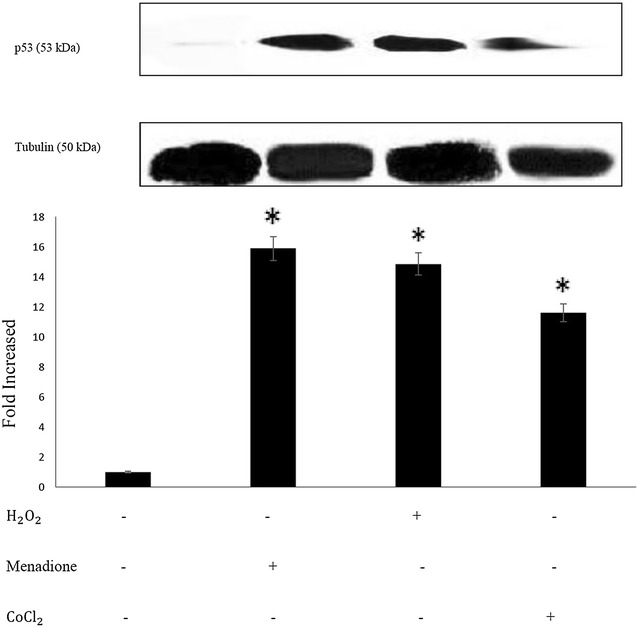
p53 activation by manadione, H_2_O_2_ and CoCl_2_. SK-N-MC cells were treated separately with 100 μM H_2_O_2_, 12 μM menadione and/or 100 μM CoCl_2_ for 24 h. P53 expression was analyzed by Western blot technique. The Intensity of each band was examined with densitometry analysis and values were normalized with respect to B-tubulin and were shown relative to the control cells. The histogram values represent the mean ± SD for three independent experiments; asterisk significantly different from untreated control cells (p < 0.05), one representative western blot is shown

In contrast to firm acceptance of hypoxia-induced HIF-1α stabilization, the accumulation (stabilization) of p53 transcriptional factor under hypoxic-to-anoxic environment is a subject of dispute in the present day literature [16, 25–28]. Under normoxia, the half-life of wild-type p53 is short mainly due to Mdm2 guided cytosolic proteasomal degradation. However, under severe oxygen depletion (< 0.2%), Mdm2 binding to the N-terminus region of p53 is apparently blocked due to the p53-phosphorylation of that domain, leading to p53 nuclear accumulation [29–31]. It has been shown, however, that hypoxia-induced p53 is transcriptionally incompetent in trans–activating many of the previously characterized target genes such as Bax, Bak, p21 [31], implying that under hypoxic environment the traditional role of p53 is lost. However, it has been shown that the traditional function could be restored under hypoxic environment by addition of a DNA damaging agent [32]. Regarding the supporting views on the presence of oxidative stress under chronic exposure to hypoxia and its subsequent influence on the cellular signaling elements, we planned to evaluate the response of p53 transcriptional factor to different types of ROS. As shown in Fig. 5, all three ROS generators, at the concentration level to induce 50% cellular apoptosis, have almost the same effect on p53 stabilization. This response is clearly different from HIF-1α response to ROS exposures (Fig. 4).

Despite the high level of p53 accumulation, the extent of CoCl_2_-induced apoptosis among the treated cells is much lower than that of the menadione and/or H_2_O_2_-treated cells (Fig. 3a, b). This could partly be attributed to the transcriptional incompetency of p53 under hypoxia (generated by CoCl_2_) as explained above.

### HIF-1α/p53 interaction in response to types of ROS

While there is universal agreement on cross-talk between HIF-1α and p53 under various stresses specifically hypoxia, the physiological significance of this interaction remains highly disputable. The primary requirement for direct and/or indirect interaction relies on the extent of expression and stabilization of each of these transcriptional factors which in turn is dependent on the intracellular oxygen tension. Evidently, at initial phase of hypoxia with an oxygen tension of around 3%, HIF-1α undergoes phosphorylation followed by its binding to HIF-1β (ARNT) and p300 with subsequent transcriptional activation. Apparently at this level of oxygen concentration, the expression of p53 is suppressed [33]. As the hypoxic condition persist for longer time and the oxygen tension drops to lower than 1%, the expression level of p53 and dephosphorylation of HIF-1α starts to increase leading to HIF-1α binding to p53 and its transcriptional inactivation [33]. Using biophysical approaches, Sanehez et al. [11] have shown that HIF-1α directly binds to p53 core domain through two distinct binding sites located at residues 530–698 (referred to as N-terminus trans activating domain, N-TAD) and residues 402–603 (termed ODD region), meaning that one HIF-1α molecule interacts with a p53 dimer.

**Fig. 6:**
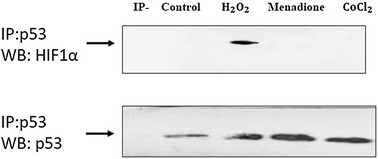
Direct interaction between HIF1-α and p53 under oxidant and imitated hypoxic condition by immunoprecipitation of p53 along with HIF1-α. SK-NM-C cells were treated with either 100 μM H_2_O_2_, 12 μM menadione or 100 μM CoCl_2_ for 24 h. Following cells lysis, immunoprecipitation was performed using p53 antibody and the immune detection was achieved using either HIF-1α (top) or p53 (bottom) antibodies. (IP-) refers to samples processed without incubation with p53 antibody

Having in mind the influence of hypoxia-induced ROS on the stabilization of both p53 and HIF-1α, we evaluated the impact of the types of ROS, under both normoxic and imitated hypoxic environments, on HIF-1α/p53 interaction following 24 h exposure of SK-N-MC cells to the IC_50_ dose of menadione (12 μM), H_2_O_2_ (100 μM) and/or CoCl_2_ (100 μM). The cell free system of each cell sample was subjected to immunoprecipitation using anti-p53 antibody followed by western blot analyses using anti-p53 and/or anti- HIF-1α antibodies to evaluate the possibility of HIF-1α and p53 interaction. As depicted in Fig. 6, under normoxic and H_2_O_2_ environments, the complex of HIF-1α/p53 has been formed. Despite the fact that the extent of p53 accumulation and also the extent of apoptosis among the cells treated with either H_2_O_2_ or menadione were almost comparable (Figs. 5 and 3, respectively), but no stable interaction between HIF-1α and p53 was registered in menadione treated cells (Fig. 6 column 3). Similarly, under hypoxic environment induced by CoCl_2_ (at 100 μM), the complex of HIF-1α/p53 did not form (Fig. 6, column 4), though p53 stabilization occurred to the same extent as under H_2_O_2_ and/or menadione treatment (Fig. 5).

Based on results presented in Figs. 2, 4 and 5, the difference in the pattern of HIF-1α and p53 communication under normoxic and non-mitochondrial dependent hypoxic environment could be attributed to two factors:

First, the difference might arise due to the types of free radicals primarily generated in the cells from H_2_O_2_, menadione and/or CoCl_2_. Secondly, and based on results presented in Fig. 4, it can be concluded that ROS dependent expression level of HIF-1α apparently plays the key role in HIF-1α/p53 communication meaning that the expression level beyond a certain limit (which requires to be determined) interrupts the interaction between HIF-1α and p53. Based on our results, expression level of HIF-1α beyond 2.5-fold has probably prevented the association between HIF-1α and p53 proteins (Figs. 4 and 6). Certainly, additional work is required to firmly approve this claim and to establish the exact range of HIF-1α level needed to initiate and/or to interrupt the communication.

Furthermore, based on results presented in Figs. 3 and 6, we cannot clearly draw a link between the extent of cellular apoptosis and the presence or absence of communication between HIF-1α and p53: Menadione and H_2_O_2_ induced almost the same extent of apoptosis among the cells while only H_2_O_2_ caused the dimerization of HIF-1α with p53 but menadione did not. On the other hand, Menadione and CoCl_2_ did not induce dimerization of HIF-1α with p53 while both induce cellular apoptosis to various extent.

In order to come up with a clearly defined physiological role for HIF-1α and p53 complex, it is essential to determine not only the transcriptional activity of each component of the complex under our experimental condition but also to identify the relevant target genes expressed by each transcriptional factor while in contact with the other component. These investigations are in progress in our lab and will be published in due course.

## Conclusions

In our study, we showed, parallel to the present literature, that intracellular ROS augmentation occurred under normoxic and/or imitated hypoxic environments leading to various degree of apoptosis among the cells. Secondly, our data clearly supported the stabilization of two of the vital transcriptional factors in charge of cellular adaption to various stresses. Thirdly, our results, in line with the literature, approved direct communication between HIF-1α and p53 under the stress environment. Fourthly, our data clearly supported the impact of types of oxygen free radicals (ROS) on modulation of direct interaction between HIF-1α and p53 proteins. We believe the capability to modulate the HIF-1α and p53 direct interaction, simply by small oxidant/antioxidant molecules, would provide a novel approach to defend against various oxidative stress damages pending the disclosure of the biological and physiological significances of HIF-1α and p53 communication.

## Experimental

### Materials

Hydrogen peroxide (H_2_O_2_) and dimethyl sulfoxide (DMSO) obtained from Merck (Darmstadt, Germany). Ethidium bromide, acridine orange, and Triton X-100 were purchased from Pharmacia LKB Biotechnology (Sweden). Ethylene diamine tetraacetic acid (EDTA), Tergitol, Anti-B-tubulin and protein G on Sepharose fast flow were from Sigma Aldrich (Germany). MTT [3-(4,5-dimethylthiazol-2,3-diphenyltetrazolium bromide], phenyl methyl sulphonyl fluoride (PMSF), 2,7-dichloroflurescein diacetate (DCFH-DA) were obtained from Molecular Probe (Eugene, Oregon, USA), Anti-HIF1α, Anti-p53 were from Santa Cruz Biotechnology, Inc. (Texas, USA). ECL kit was purchased from Amersham-Pharmacia (Piscataway, NJ, USA). Mouse/Rabbit horse radish peroxidase-conjugated secondary antibodies were from Bio Source (Nivelles, Belgium). The cell culture medium (RPMI-1640), penicillin–streptomycin, and fetal bovine serum (FBS) were purchased from Gibco BRL (Life technology, Paisely, Scotland). The culture plates were from Nunc (Denmark). Human SK-N-MC neuroblastoma cells were obtained from Pasteur institute (Tehran, Iran).

### Cell culture

The SK-N-MC cells were cultured in RPMI-1640 medium supplemented with 10% (v/v) fetal bovine serum (FBS) and 1% streptomycin-penicillin. Cells were maintained at 37 °C in the incubator with 5% CO_2_-humidified atmosphere.

### Treatments

Cells were treated with H_2_O_2_, menadione and/or Cobalt Chloride at different doses. H_2_O_2_ was diluted with deionized water and then its concentration was determined by its absorption at 240 nm. Menadione was dissolved in dimethyl sulfoxide (DMSO) and then diluted with the culture medium to the desired concentration. The concentration of DMSO in the culture medium kept lower than 0.1%. Cobalt Chloride was dissolved in deionized water to the desired concentration.

### Hypoxia induction

Imitation of hypoxic condition was achieved by the exposure of the cells to Cobalt Chloride solution. Cells were seeded at a density of 7.5 × 10^5^ cells/ml and incubated for 24 h at 37 °C. Then, they were treated with 100 μM Cobalt Chloride for different time intervals. The cells’ viability was determined by MTT assay to establish the IC_50_. The remaining experiments were done at a CoCl_2_ Concentration below its IC_50_.

### Cell viability measurement

Cell viability was assessed using the MTT assay which determines the extent of mitochondrial dehydrogenase activity in living cells. SK-N-MC cells were seeded into 96-well plates at a density of 2.5 × 10^4^ cells/well in 200 μl of medium. After 12 h, cells were treated with different concentration of H_2_O_2_, menadione and/or CoCl_2_ and incubated for 24 h. Then, 10 μl of MTT reagent was added to each well, with a final concentration of about 4 mg/ml, followed by the cells incubation for 4 h. Then, the medium was discarded and 100 μl DMSO was added to each well. The formazan dye crystals were solubilized at room temperature and the absorbance at 570 nm was measured with an ELISA reader. Results were presented as the percentage of MTT reduction, assuming that the absorbance of the control cells was 100%.

### Determination of reactive oxygen species

The reactive oxygen content of the cells was measured by DCF-DA probe [34]. Cells were seeded at the stated density. Then, cells were treated with the desired concentrations of each of the drug (menadione, Cobalt Chloride and/or H_2_O_2_ (. After 24 h, cells were collected and washed twice with PBS. Then, equal cell number of each sample was incubated with DCFH-DA probe in a final concentration of 10 μM for 30 min. Finally, the fluorescent intensity was monitored at 485 nm.

### Fluorescence microscopy evaluation of the apoptotic cells

Dual ethidium bromide/acridine orange (EtBr/Ao) cell staining was used to study apoptosis. In this approach, the normal cells get stained green while the apoptotic cells are stained orange. Treated cells were collected and washed twice with PBS, then, the staining mixture containing acridine orange (1 μg/ml) and ethidium bromide (1 μg/ml) at 1:1 ratio was added to the cells. The stained cells were evaluated by an Axoscope 2 plus fluorescence microscope from Zeiss (Gena, Germany).

### Immunoblot evaluations

The control and each of the oxidant-treated cell samples were collected and lysed using the lysis buffer (100 mM Tris–HCl, pH = 7.5, 1% Triton X-100, 1% sodium dodecyl sulphate (SDS), 100 mM NaCl, 2 mM ethylenediamine tetraacetate, 3 mM sodium orthovanadate, 1 mM NaF, 1 mM phenyl methyl sulfonyl fluoride, 1 μg/ml pepstatine and 1 mM DTT, 10 μg/ml leupeptine. Total protein concentration was measured by the Lowry’s method and 100 μg protein content of each sample was loaded on a 10 or 12% SDS-PAGE. Afterward, the resolved proteins were electro blotted to a pieces of PVDF membrane for 2 h. The membrane was then blocked by 5% (w/v) non-fat dry milk/TBST (Tris-base 20 mM, NaCl 137 mM and Tween-20.5%) for 90 min at room temperature. The blocked membrane was incubated with the diluted primary antibody following the manufacture’s instruction for an overnight at 4 °C and after three time washing, each membrane was incubated with the anti-rabbit or anti-mouse horse radish peroxidase (HRP)-conjugated secondary antibody at a predetermined concentration for 90 min at room temperature. The proteins were detected by an ECL kit according to the manufacturer’s instruction.

### Immunoprecipitation

For immunoprecipitation, the cells were seeded at the appropriate density and treated by the desired concentrations of menadione, Cobalt Chloride and/or H_2_O_2_ for 24 h. Then, the cells were washed twice with PBS, lysed with RIPA buffer containing 50 mM Tris–HCl (pH 8), 150 mM NaCl, 1% NP-40, 0.5% sodium deoxy cholate, 0.1% SDS. Afterwards, equal protein content (200 μg) of each sample was incubated with recommended amount of anti-HIF1-α protein under low agitation overnight at 4 °C, subsequently the slurry was mixed with appropriate amount of protein G-agarose fast flow beads and incubated at 4 °C for 12 h. After this step, supernatant was discarded and the beads were washed 3 times with the washing buffer (50 mM Tris–HCl, pH 8, 150 mM NaCl, 1% NP-40). Loading buffer was added and samples were boiled for 5 min. Finally, an aliquot of each sample was loaded on a SDS-PAGE gel and the band detection was achieved by ECL detection technique.

### Statistical analysis

Data were expressed as percent of values of untreated control cells, and each value represents the mean ± SD (n = 3). For IC50, MTT data were analyzed by compusyn software. For western blots, data were normalized with respect to tubulin by image j software and the significant differences between the means of the treated and untreated cells were calculated by unpaired student’s t test and P < 0.05 was considered significant.
